# National Insurance in Germany and Great Britain

**Published:** 1912-10-05

**Authors:** 


### The Hospital

The Workers' Newspaper of Administrative Medicine and Institutional Life, Administration, National Insurance and Health.

No. 1370, Vol. LIII. SATURDAY, OCTOBER 5, 1912. PRICE ONE PENNY.

### NATIONAL INSURANCE IN GERMANY AND GREAT BRITAIN.

Being conscious of the many misapprehensions which prevail in England concerning national insurance in Germany, we have made it our business to examine into the various questions involved, and for this purpose have spent some weeks there. Our commissioner's tour of inspection, though not without difficulties, was full of interest, and we propose to publish the results in a series of articles, which will appear week by week. In Germany national insurance was begun by experimental attempts to find a firm working basis upon which this great and much-needed legislation could rest. Possibly it may be startling for British readers to learn that at the present moment Germany has no system of national insurance. National or Imperial insurance will not come into force in Germany until January 1, 1913. At the present time what is known as national insurance, which was begun experimentally in 1885, has gradually been built up and now extends throughout each of the States which collectively constitute the Germany of to-day.

In some States the system of insurance is worked under the supervision and direction of the State or Government department. In certain cities State insurance, we were informed, is under the control of the municipality, which is responsible for the supervision of sickness and accident insurance. In both cases, whether it be the State or the municipality that directs the insurance business, the rule is that should there be a deficiency in any year in the funds of the Kassa, or approved society, the State or municipality will advance the necessary funds to make good the deficiency. Should such a case occur the State or municipal authorities would recoup themselves by insisting upon the societies concerned increasing the levies upon their members to a sum sufficient to make good the deficiency in question. There is in Germany an Imperial system of invalid or old-age pension insurance, but that is quite distinct in itself and has no connection with sickness and accident insurance. The great distinction between the English and German plan of invalid or old-age pension insurance is that that in Germany is properly organised upon a contributory basis, whilst in England it is non-contributory. Anyone who really studies the two systems in the light of the experience of Germany will come to the conclusion that it is a misfortune to the nation that England should have a non-contributory old-age pension system at the present time. It is bad business for the State and for the individual citizen.

When we come to the question of medical and hospital benefits under the German system the grave injustice, impracticability, and dangers of the British National Insurance Act as it stands become strikingly apparent. All the hospitals throughout Germany, with a few exceptions, are either municipal hospitals, where any deficiency is made good by the Town Council of the town where they are situated, or clinical hospitals, like the Royal Charité Hospital in Berlin, where the State or Government exercise supreme control and provide the necessary funds for the adequate carrying on of the work. The necessary funds are confined to the sum represented by any deficiency which there may be in a given year between the total sum derived from the patients' payments and the actual cost of maintaining each hospital. Both in the Government and in the town hospitals of Germany all patients pay so much per diem for their maintenance. In the majority of the hospitals there is more than one class of pay patient, and the fees paid vary from 2.50 marks to 10 or even more marks per diem, according to the accommodation required and the class to which the patient belongs. In this way the bulk of the money for the support of the hospitals is obtained. Again, all insured persons admitted to the hospital are required to pay from 2.50 marks to 3.50 marks for their care and treatment. This money is received by the hospitals from the approved society to which each patient may belong, and such payments are made weekly to the hospitals. In some hospitals the system is to require a deposit of 50 marks by the patient or his representatives on admission. Under this system there are no losses by the hospitals, which experience little or no difficulty in collecting their revenues, being the payments of individual patients

B

218866

or payments by the approved societies on behalf of their members admitted to the hospitals.

We have advocated a plan for adoption by the general hospitals in Great Britain in agreement with the Insurance Commissioners which would maintain the voluntary principle of hospital support and at the same time materially aid the Government by reducing the expenditure upon the cost of hospital provision to a minimum. It would obviate the liability which must at present attach to the Government of providing large capital sums for hospital purposes. Our plan is suited to the habits and requirements of the population of these islands and would be most economical and satisfactory in practice. It is not our purpose to-day, however, to attempt to elaborate the details or to explain fully the plan upon which the system has been gradually built up and now works so successfully in Germany. The whole question is one of the first importance, however, to millions of people in these islands. There can be no doubt that our publication of accurate data based upon the working of popular insurance in Germany must contribute materially to the speedy introduction of an Amending Act. Thus the present perils by which national insurance is threatened in England can, by an enactment based upon precise knowledge and indisputable data, be effectively removed.

